# Homogenous TP53mut-associated tumor biology across mutation and cancer types revealed by transcriptome analysis

**DOI:** 10.1038/s41420-023-01413-1

**Published:** 2023-04-14

**Authors:** Eva Romanovsky, Klaus Kluck, Iordanis Ourailidis, Michael Menzel, Susanne Beck, Markus Ball, Daniel Kazdal, Petros Christopoulos, Peter Schirmacher, Thorsten Stiewe, Albrecht Stenzinger, Jan Budczies

**Affiliations:** 1 Institute of Pathology, Heidelberg University Hospital, 69120 Heidelberg, Germany; 2 Center for Personalized Medicine (ZPM) Heidelberg, 69120 Heidelberg, Germany; 3 Department of Thoracic Oncology, Thoraxklinik and National Center for Tumor Diseases (NCT) Heidelberg, member of the German Center for Lung Research (DZL), Heidelberg, Germany; 4 German Cancer Consortium (DKTK), Heidelberg partner site, Heidelberg, Germany; 5 Institute of Molecular Oncology, member of the German Center for Lung Research (DZL), Philipps-University, 35037 Marburg, Germany

**Keywords:** Diagnostic markers, Prognostic markers

## Abstract

*TP53* is the most frequently mutated gene in human cancer. While no *TP53*-targeting drugs have been approved in the USA or Europe so far, preclinical and clinical studies are underway to investigate targeting of specific or all *TP53* mutations, for example, by restoration of the functionality of mutated *TP53* (TP53mut) or protecting wildtype *TP53* (TP53wt) from negative regulation. We performed a comprehensive mRNA expression analysis in 24 cancer types of TCGA to extract (i) a consensus expression signature shared across *TP53* mutation types and cancer types, (ii) differential gene expression patterns between tumors harboring different *TP53* mutation types such as loss of function, gain of function or dominant-negative mutations, and (iii) cancer-type-specific patterns of gene expression and immune infiltration. Analysis of mutational hotspots revealed both similarities across cancer types and cancer type-specific hotspots. Underlying ubiquitous and cancer type-specific mutational processes with the associated mutational signatures contributed to explaining this observation. Virtually no genes were differentially expressed between tumors harboring different *TP53* mutation types, while hundreds of genes were over- and underexpressed in TP53mut compared to TP53wt tumors. A consensus list included 178 genes that were overexpressed and 32 genes that were underexpressed in the TP53mut tumors of at least 16 of the investigated 24 cancer types. In an association analysis of immune infiltration with *TP53* mutations in 32 cancer subtypes, decreased immune infiltration was observed in six subtypes, increased infiltration in two subtypes, a mixed pattern of decreased and increased immune cell populations in four subtypes, while immune infiltration was not associated with *TP53* status in 20 subtypes. The analysis of a large cohort of human tumors complements results from experimental studies and supports the view that *TP53* mutations should be further evaluated as predictive markers for immunotherapy and targeted therapies.

## Introduction

The “guardian of the genome” *TP53* is the most frequently mutated gene in malignant tumors [1, 2]. While *TP53* mutations can be found in most cancer types, their prevalence varies strongly between different entities [3, 4]. The vast majority of *TP53* mutations are located in the DNA-binding domain (DBD), whereas *TP53* mutations in other regions are found at lower frequency [5–10]. *TP53* mutations accumulate in mutational hotspots, including the most frequently mutated amino acids R175, R248, and R273 [11, 12].

The diverse spectrum of *TP53* mutations has motivated numerous studies to uncover the effect of mutated p53 proteins using cultured cells, animal models, and molecular profiling of human tumors [10, 13–17]. Insights from these studies support four different mechanisms of how *TP53* mutations contribute to malignant growth: (i) loss of function (LOF) mutations impairing the tumor suppressor functions of p53, including its action as a transcription factor [18, 19], (ii) gain of function mutations (GOF) adding new oncogenic functions [20], (iii) impact of mutated p53 as a dominant-negative (DN) inhibitor of the wildtype p53 protein [21], (iv) action through separation of functions that is loss of some of the functions of the wildtype p53 protein, while other functions are retained. These four possible mechanisms do not exclude each other, e.g., oncogenic *TP53* mutations are frequently accompanied by at least partial LOF [22].

Among the four possibilities, the evidence for action of p53 through total or partial LOF is very high. Numerous studies have demonstrated the connection of *TP53* LOF mutations and failure of the mutated p53 protein to induce its classical transcriptional targets including the cell cycle regulator *CDKN1A* (p21) [23–26]. Examples of mutant *TP53* acting through partial LOF include mutations located in the acidic transactivation domains resulting in a truncated protein that retains the ability to induce apoptosis [27] and mutations in the DBD such as E180R and R181C/H/L being defective only in the induction of apoptosis but still able to induce cell cycle arrest [22].

Distinct hotspots in the distribution of *TP53* mutations suggest a positive selection pressure on specific mutations driven by specific functional gains and corresponding growth advantages. TP53mut GOF activity was first reported in the early 1990s by the investigation of in vitro and in vivo models of ectopically expressed TP53mut in *TP53* null cells that allowed separation of GOF from DN activity [28, 29]. Since then, GOF mutations have been reported in many studies and associated with tumor growth, invasion, metastasis, and poor prognosis [7, 30]. DN activity of *TP53* missense mutations without evidence of GOF capacity has been observed analyzing in vitro and in vivo models of acute myeloid leukemia (AML) [31]. Concordant mutational spectra were observed comparing normal cells and carcinoma of the skin and the esophagus, opposing the view of a selective advantage of specific *TP53* GOF mutations over others [32, 33].

Although *TP53* mutations are highly prevalent in many cancer types, *TP53*-targeting drugs have not yet been approved in the USA or Europe. In this context, *TP53* mutational diversity and differently acting *TP53* mutation types are a hurdle that could be overcome by targeting specific *TP53* alterations. For example, a predominant LOF effect should be addressed by restoration of wildtype p53 protein expression, while a predominant DN or GOF effect should be addressed by inhibition of mutant p53 protein levels [14]. Earlier this year, a phase I clinical study showed promising results for targeting Y220C with a small molecule structural corrector to restore the wildtype conformation [34]. As a basis for such treatment approaches, further investigation of the spectrum of *TP53* mutations and the corresponding pathogenic mechanisms are warranted.

To contribute to fill this gap, we analyzed the impact of *TP53* mutations on tumor biology in 8331 tumors of 24 cancer types from TCGA. Building on and expanding previous gene expression studies [35, 36], our analysis focused on the following novel aspects: (i) the influence of *TP53* mutation types - either of specific variants or variant classes including LOF, GOF, or DN mutations - on gene expression patterns, (ii) separation of common TP53mut-associated expression changes that are shared between many cancer types and specific expression changes observed only in a single or in a few cancer types, and (iii) changes in the immune tumor microenvironment (TME) associated with *TP53* mutations.

## Results

A total of 8331 tumors and 24 cancer types from TCGA were included in the study. The cohort was divided into tumors harboring non-synonymous *TP53* mutations in the coding sequence or at splice sites (TP53mut tumors, *n* = 3447) and tumors without such mutations (TP53wt tumors, *n* = 4884).

### Analysis of *TP53* mutation hotspots

**Fig. 1:**
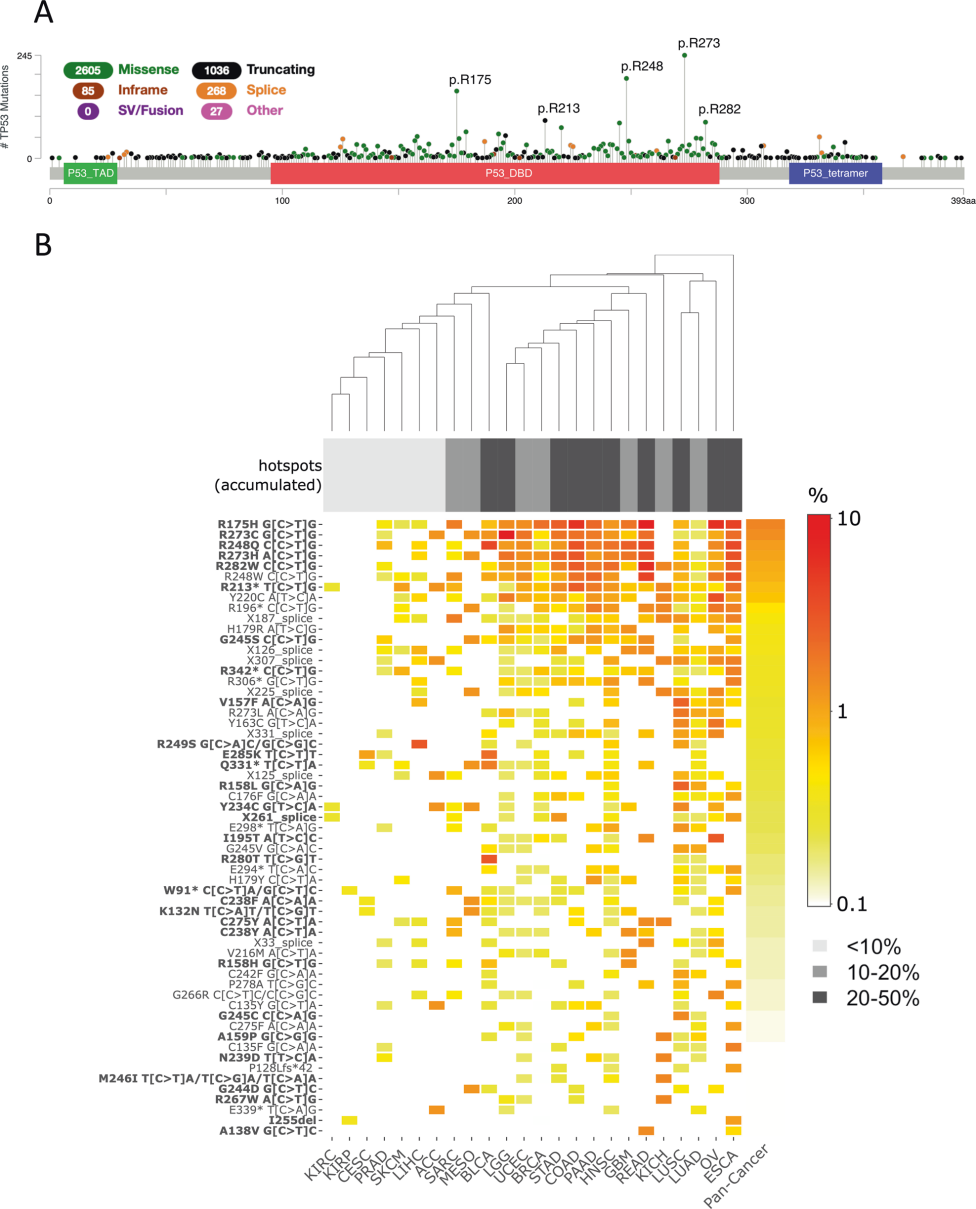
Hotspots of TP53 mutations. **A** Lollipop diagram showing the prevalence of *TP53* mutations in the TCGA cohort across cancer types. **B** Heatmap showing the prevalence of *TP53* mutations in specific cancer types of the TCGA cohort. All mutations that are prevalent with a frequency of at least 1% in at least one cancer type and are mutated in at least two tumors of at least one cancer type are included. Hotspot mutations with significantly different prevalences in different cancer types are in bold.

We detected 4021 individual *TP53* mutations in the study cohort, corresponding to 926 different variants (Fig. 1A). The most frequent mutation types were: missense mutations (65%), truncating mutations (26%), and splice site mutations (7%). The most frequently affected mutation hotspots p.R273 (*n* = 250), p.R248 (*n* = 201), p.R175 (*n* = 165), p.R213 (*n* = 96), and p.R282 (*n* = 90) were all located in the DBD. In codon 273 the mutations p.R273C (46%) and p.R273H (37%) were most prevalent, in codon 248 the mutations p.R248Q (54%) and p.R248W (39%), while in codon 175 the mutation p.R175H (89%) was by far most prevalent.

Next, we compiled a list of *TP53* hotspots that were recurrent with an incidence of at least 1% in at least one of the cancer types (Fig. 1B). Altogether, there were 59 mutations, including 40 missense mutations, 11 truncating mutations, and eight splice site mutations. Most of the recurrent mutations were detected in many cancer types: 45% of the hotpot mutations were detected in at least ten cancer types, while 88% of the hotspot mutations were detected in at least five cancer types. The seven most prevalent hotspot mutations (top of the heatmap) were all transitions of the type CG > TG. These represent the footprint of mutational signature SBS1 and the clock-like mutational process that is driven by spontaneous deamination of 5-methylcytosine and active in all cancer types [37]. The high prevalence of the top mutational hotspots in most of the cancer types is in line with the ubiquitous activity of this mutational process.

The prevalence of 31 mutations (53%) was significantly different between TP53mut tumors of different cancer types (bold in Fig. 1 and Suppl. Table S2). The following mutations showed the strongest enrichments in specific cancer types: R175H contributed to *TP53* mutations with 10% (95% CI: 6–15%) in COAD and 11% (5–18%) in READ compared to 3.7% (3.1–4.4%) in the pan-cancer cohort. R273C contributed with 21% (16–27%) in LGG compared to 3.3% (2.7–3.9%) in the pan-cancer cohort. R249S contributed with 10% (5.2–17%) in LIHC compared to 0.6% (0.4–1%) in the pan-cancer cohort. These cancer-specific differences in the prevalence of *TP53* mutations can be explained, at least in part, by the differential activity of mutational signatures in different cancer types. For example, the mutations R249S with a GCC > CGC transversion that was highly prevalent in LIHC is a characteristic of the mutational signature SBS24 that has been linked to exposure to liver toxic aflatoxins [38]. Furthermore, the mutations V157F and R158L were highly prevalent in smoking-associated cancer types LUAD and LUSC. Both mutations are generated by an A > C transversion, characteristic of the tobacco smoking-associated mutational signature SBS4.

### Gene expression patterns associated with *TP53* mutation types

We investigated the hypothesis that functionally different *TP53* mutations may produce distinct gene expression patterns. To this end, we grouped the tumors according to *TP53* mutation type resulting in 2050 (59%) tumors with LOF, 1208 (35%) with GOF, 1470 (43%) tumors with DN, and 1788 (52%) tumors with non-DN mutations (Suppl. Fig. S1 and Suppl. Table S3). The two classification systems had a large overlap: 1086 (90%) of the tumors in the class GOF were also in the class DN, and 1666 (81%) of the tumors in the class LOF were also in the class non-DN.

**Fig. 2:**
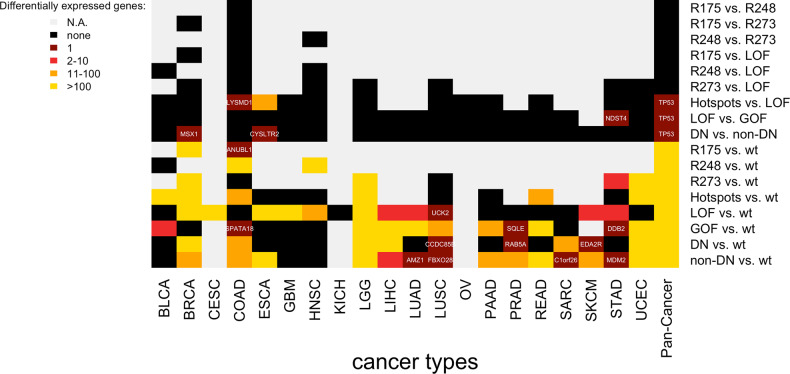
Gene expression patterns associated with GOF, LOF, DN, and non-DN *TP53* mutations. For each analysis in a specific cancer type, 15 samples of a *TP53* mutation class were compared to 15 samples of another mutation class. For the pan-cancer analysis, the results for specific cancer type were summarized using Fisher’s method. None = no significantly expressed genes detected, N.A. = analysis not possible (insufficient number of samples). Hotspots = pool of the 10 most abundant *TP53* missense mutations in the study cohort (R175H, R273C/H, R248Q/W, R282W, Y220C, G45S, H179R, and V157F).

We analyzed differential gene expression between (i) tumors with mutations in codons 175, 248, and 273, (ii) tumors with mutations in codon 175 and LOF mutations, (iii) tumors with mutations in codon 248 and LOF mutations, (iv) tumors with mutations in codon 237 and LOF mutations, (v) tumors with top hotspot mutations (pool of the 10 most abundant missense mutations) and LOF mutations, (vi) tumors with LOF and GOF mutations, and (vii) tumors with DN and non-DN mutations (Fig. 2 and Suppl. Fig. S2). These analyses were contrasted by differential expression analyses comparing tumors with different types of *TP53* mutations to TP53wt tumors. To ensure comparability of the gene expression analyses, we always compared groups of 15 tumors to groups of 15 tumors using random subsampling. For the first type of analysis (analyses i-vii comparing TP53mut subtypes), we almost never observed more than one differentially expressed gene and not a single differentially expressed gene in the majority (92%) of the analyses. For the second type of analysis (TP53mut tumors vs. TP53wt tumors), we detected more than ten differentially expressed genes in 48 (48%) of the analyses, 2 to 10 differentially expressed genes in seven (7%) of the analyses, and only one or none differentially expressed gene in the remaining 45 (45%) analyses. When summarizing significances over the 24 analyzed cancer types (column “pan-cancer”), not a single differentially expressed gene except *TP53* was detected for the analyses of the first type, while more than 100 differentially expressed genes were detected for each of the analyses of the second type. Similar results were observed when we subsampled to larger (*n* = 20) or smaller (*n* = 10) groups of tumors (Suppl. Fig. S2).

In summary, we detected many differentially expressed genes between TP53mut and TP53wt tumors. However, we did not find gene expression patterns characteristic for the subclasses of LOF, GOF, and DN mutations in any of the cancer types.

### Pan-cancer consensus gene expression pattern of TP53mut tumors

A consensus list of 210 differentially expressed genes included all genes that were differentially expressed (raw *p* < 0.05) between TP53mut and TP53wt tumors in at least two-thirds (*n* = 16) of the cancer types (Suppl. Table S4). We summarized the p-values of each gene across the cancer types resulting in *p* < 1.0E-28 for the genes in the consensus list, which was far below the Bonferroni threshold. Overall, 178 (85%) genes were overexpressed in TP53mut tumors in the majority of cancer types, while the remaining 32 genes (15%) were underexpressed in the majority of cancer types. We analyzed the lists of over- and underexpressed genes for enrichment in a catalog of 186 KEGG pathways and six *TP53*-related gene lists provided by Fischer et al. [39]. We found 11 significantly enriched categories for overexpressed genes and eight significantly enriched categories for underexpressed genes (Table 1). Of the overexpressed genes, 66% were the targets of dimerization partner, RB-like, E2F and multi-vulval class B (DREAM) complex (enrichment FC = 14.8), 29% were annotated to the G2/M phase of the cell cycle (enrichment FC = 24.4), and 7% were annotated to the G1/S phase of the cell cycle (enrichment FC = 7.8). Of the underexpressed genes, 53% were direct p53 targets (enrichment FC = 34.4), 28% were annotated to the KEGG p53 signaling pathway (enrichment FC = 79.1), and 9% were annotated to the KEGG apoptosis pathway (enrichment FC = 19.9).

**Table 1:** Functional analysis of the pan-cancer consensus list of differentially expressed genes between TP53mut and TP53wt tumors.

A				
Category	Number of genes in category	Overexpressed genes in category (%)	Enrichment FC	*p* value
FISCHER_DREAM_TARGETS	821	65.7	14.8	4.9e-119
FISCHER_G2_M_CELL_CYCLE	218	28.7	24.4	1.1e-57
KEGG_CELL_CYCLE	118	11.2	17.6	1.2e-19
KEGG_DNA_REPLICATION	36	5.1	26	4.2e-11
KEGG_OOCYTE_MEIOSIS	110	6.2	10.4	8.5e-09
FISCHER_G1_S_CELL_CYCLE	173	7.3	7.8	1.2e-08
KEGG_PROGESTERONE_MEDIATED_OOCYTE_MATURATION	85	4.5	9.8	1.6e-06
KEGG_P53_SIGNALING_PATHWAY	65	3.9	11.2	2.9e-06
RIEGE_DELTANP63_DIRECT_TARGETS_UP	128	3.9	5.7	0.00023
KEGG_HOMOLOGOUS_RECOMBINATION	24	1.7	13	0.0015
KEGG_BASE_EXCISION_REPAIR	33	1.7	9.5	0.0038

B				
Category	Number of genes in category	Underexpressed genes in category (%)	Enrichment FC	*p* value
FISCHER_DIRECT_P53_TARGETS_META_ANALYSIS	282	53.1	34.4	2.3e-23
KEGG_P53_SIGNALING_PATHWAY	65	28.1	79.1	1.2e-15
KEGG_APOPTOSIS	86	9.4	19.9	0.00045
KEGG_RIBOSOME	87	9.4	19.7	0.00046
KEGG_BLADDER_CANCER	40	6.3	28.5	0.0022
KEGG_PATHWAYS_IN_CANCER	320	12.5	7.1	0.0022
KEGG_NUCLEOTIDE_EXCISION_REPAIR	44	6.3	26	0.0027
KEGG_HUNTINGTONS_DISEASE	160	9.4	10.7	0.0027

We performed a gene set enrichment analysis with respect to the KEGG pathways and categories published by Fischer et al. [39] A, Significantly enriched categories in the set of overexpressed genes. B, Significantly enriched categories in the set of underexpressed genes.

**Fig. 3:**
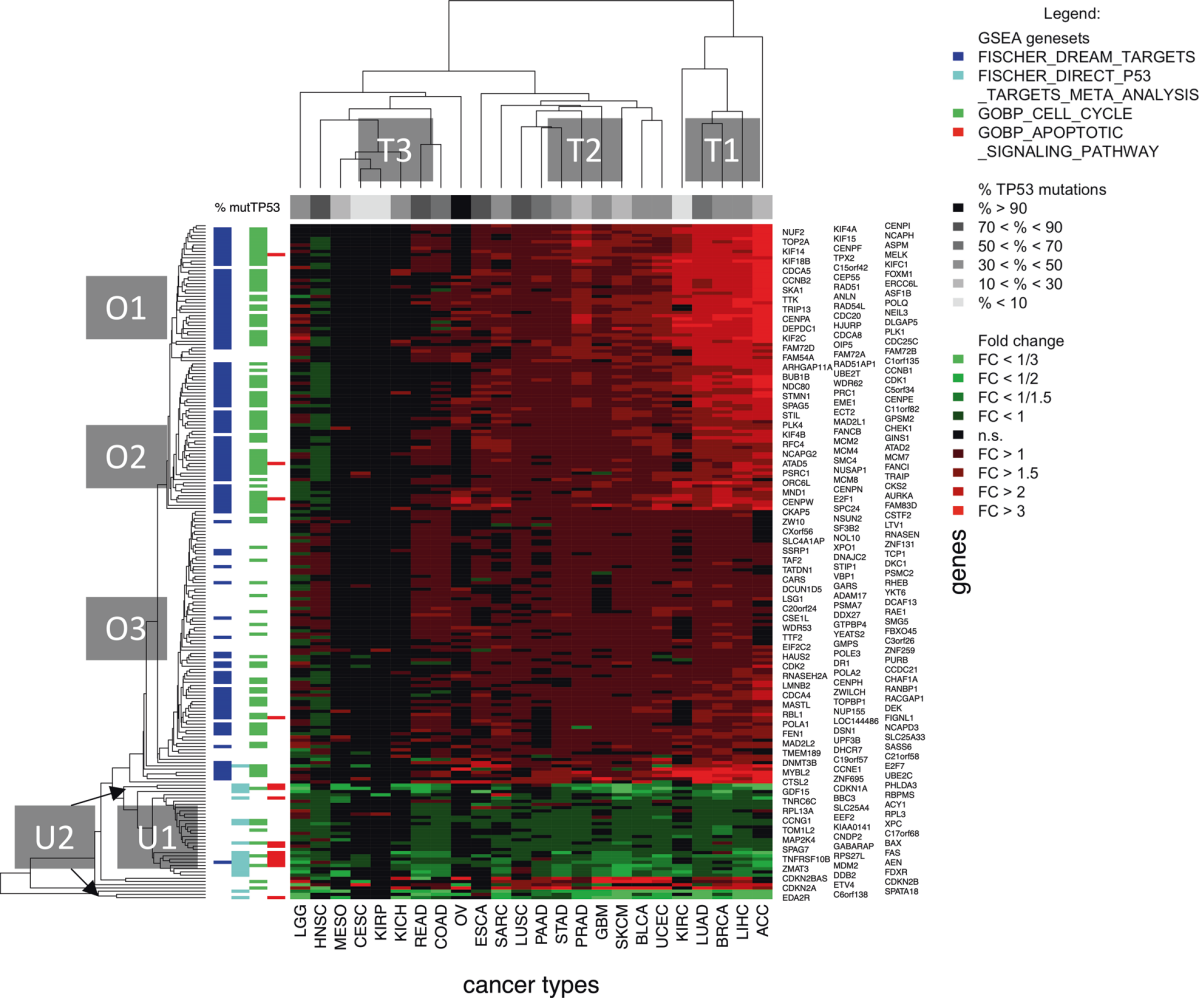
Heatmap analysis of the consensus list of 210 differentially expressed genes. Genes that were significantly (raw *p* < 0.05) differentially expressed between TP53mut and TP53wt tumors in at least 16 of the 24 cancer types were included in the consensus list. Significant FCs (FDR = 10%) between TP53mut and TP53wt tumors are coded in red or green.

A heatmap of FCs of the 210 genes in the 24 cancer types showed a high degree of consistency in the direction of expression changes across the cancer types (Fig. 3). Gene clustering resulted in the following six clusters: genes with strong (O1), moderate (O2), and weak overexpression in TP53mut tumors (O3), as well as one cluster with weak (U1) and two clusters with strong underexpression in TP53mut tumors (U2). We annotated the genes of the consensus list to the following categories of the MSigDB: Targets of the DREAM complex, direct targets of *TP53*, cell cycle, and apoptosis. Many of the overexpressed genes (clusters O1, O2, and O3) were targets of the DREAM complex (66% of the genes) and related to the GO category cell cycle progression (57%). Many of the underexpressed genes (cluster U1 and U2) were direct p53 targets (53%) and related to the GO category apoptosis (34%). *CDKN1A* (p21), *SPATA18*, *EDA2R*, *PHLDA3*, and *C6orf138* in cluster U2 showed a strongly diminished gene expression in the TP53mut tumors for most of the cancer types. In line with this observation, *CDKN1A*, *SPATA18*, *EDA2R*, and *PHLDA3* are known as direct p53 targets regulated by the binding of p53 to the promoter sequence [40, 41].

Cancer types were grouped into a cluster of five cancer types showing strong overexpression of gene clusters O1/O2/O3 in TP53mut tumors (T1: ACC, BRCA, LUAD, LIHC, and KIRC), a cluster of ten cancer types showing moderate overexpression (T2: UCEC, ESCA, SKCM, GBM, BLCA, PRAD, SARC, STAD, PAAD, and LUSC), and a cluster of nine cancer types showing very low or no overexpression of these genes (T3: KIRP, LGG, HNSC, CESC, COAD, READ, KICH, MESO, and OV). Overexpression of the gene clusters O1/O2/O3 that were enriched for indirect, p21-mediated targets corresponded to underexpression of the gene clusters U1/U2 that were enriched for direct p53 targets. Clusters T1, T2, and T3 were not associated with different prevalence of *TP53* mutations in cancer types (*p* = 0.33). In some instances, different patterns of differential expression were observed in tumors of the same organ site. For example, TP53mut LUAD (cluster T1) showed strong overexpression of O1/O2/O3, while TP53mut LUSC (cluster T2) showed only moderate overexpression. TP53mut KIRC (cluster T1) showed moderate overexpression, while TP53mut KICH and KIRP (cluster T3) did not show any overexpression of the gene clusters O1/O2/O3.

We analyzed the prognostic relevance of the genes in the consensus list and compared the hazard ratios (HRs) associated with differential gene expression (above median vs. below median) with the ones associated with *TP53* status (mut vs. wt, Suppl. Fig. S3). For many cancer types (ACC, KICH, KIRC, LGG, LIHC, LUAD, MESO, PAAD, PARD, SARC, and UCEC), patients with TP53mut tumors, tumors with high expression of cell cycle genes, as well as tumors with low expression of direct p53 targets, had poorer prognosis. By contrast, for some cancer types, including CESC, COAD, GBM, READ, and STAD, patients with TP53mut tumors and tumors with high expression of cell cycle genes had better prognosis. Of note, altogether the expression levels of the genes in the consensus list were prognostic for more cancer types than the TP53mut status.

### Cancer-type-specific gene expression patterns of TP53mut tumors

**Fig. 4:**
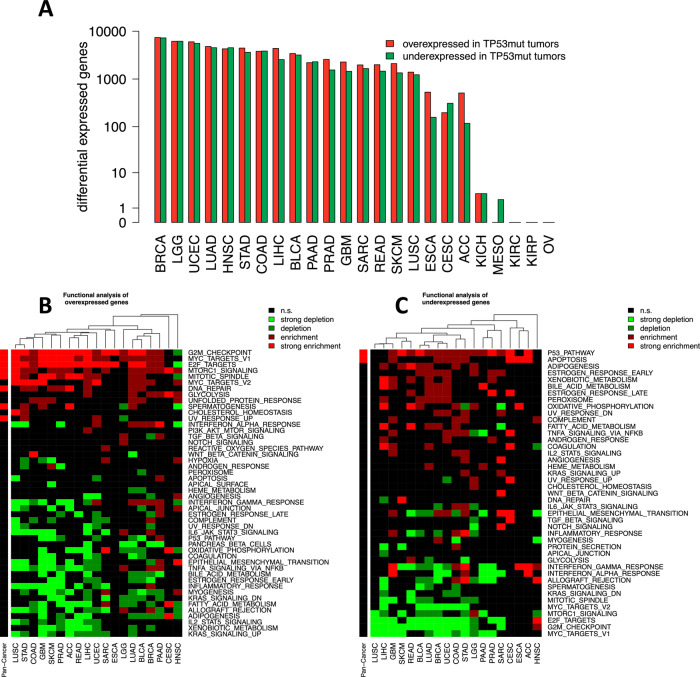
Differential gene expression and functional analysis in specific cancer types. **A** Numbers of significantly (FDR = 10%) differentially expressed genes between TP53mut and TP53wt tumors for 24 specific cancer types. **B** Significantly enriched categories of the hallmark catalog in the set of overexpressed genes. **C** Same as **B**, but for the set of underexpressed genes. Significantly enriched or depleted gene sets are shown in red or green. Multiple testing correction (FDR = 10%) was performed, including both cancer types and analyzed categories (20 ×50 hypotheses). Enrichment FC = proportion of the genes in the gene set annotated to the hallmark / proportion of the genes in the genome annotated to the hallmark.

We analyzed differential expression separately in each of the 24 cancer types (FDR = 10%, Fig. 4A). Differentially expressed genes were detected in 21 cancer types (all except KIRC, KIRP, and OV). WE analyzed the lists of over- and underexpressed genes for enrichment or depletion of the 50 hallmark gene sets of MSigDB (Fig. 4B, C). The hallmarks ‘G2M_CHECKPOINT’, ‘E2F_TARGETS’, and ‘MYC_TARGETS_V1’ were enriched in the overexpressed genes of 17, 16, and 16 cancer types, in line with enhanced proliferation in TP53mut tumors (Fig. 4B). At the same time, these categories were depleted the underexpressed genes of 13, 13, and 12 cancer types (Fig. 4C). Overexpressed genes were enriched for ‘MTORC1_SIGNALING’ in 14 cancer types and for ‘MITOTIC SPINDLE’ in 11 cancer types. Overexpressed genes in a minority of cancer types were enriched for ‘DNA REPAIR’ (8 cancer types), ‘GLYCOLYSIS’ (6 cancer types: LIHC, LUAD, BRCA, PAAD, CESC, and HNSC), and ‘UNFOLDED PROTEIN RESPONSE (6 cancer types: COAD, GBM, SKCM, LUAD, BLCA, and HNSC). The hallmarks ‘INTERFERON_ALPHA_RESPONSE’ and ‘INTERFERON_GAMMA_RESPONSE’ were enriched in the overexpressed genes of five cancer types (UCEC, LUAD, BLCA, BRCA, and PAAD), but depleted in respectively four and seven cancer types. Underexpressed genes of 15 cancer types were enriched for ‘P53_PATHWAY’ and of eight cancer types for ‘APOPTOSIS’ in line with a failure of mutated *TP53* to bind to the regulatory DNA sequences of its direct target genes that is predicted for most of the *TP53* mutations.

The enrichment of genes of the glycolysis pathway in the set of overexpressed genes in six cancer types suggests a stronger Warburg effect in the TP53mut tumors of these cancer types. Among the 168 overexpressed genes in the glycolysis pathway, six genes were direct targets of p53 (*ABCB6*, *IER3*, *GPC1*, *GPR87*, *NDUFV3*, and *VCAN*).

Altogether, we observed similar patterns of enrichment and depletion across many cancer types, but also activation and deactivation of specific cancer hallmarks in individual cancer types. For example, for PAAD the genes overexpressed in TP53mut tumors were enriched for the gene sets ‘P53_PATHWAY’ and ‘APOPTOSIS’ in contrast to an enrichment of these gene sets in the underexpressed genes for most other cancer types. For HNSC, the overexpressed genes were depleted for the gene sets ‘G2M_CHECKPOINT’ and ‘E2F_TARGETS’ in contrast to the enrichment of these gene sets for most other cancer types.

### Pathways analysis of TP53mut-associated gene expression patterns

**Fig. 5:**
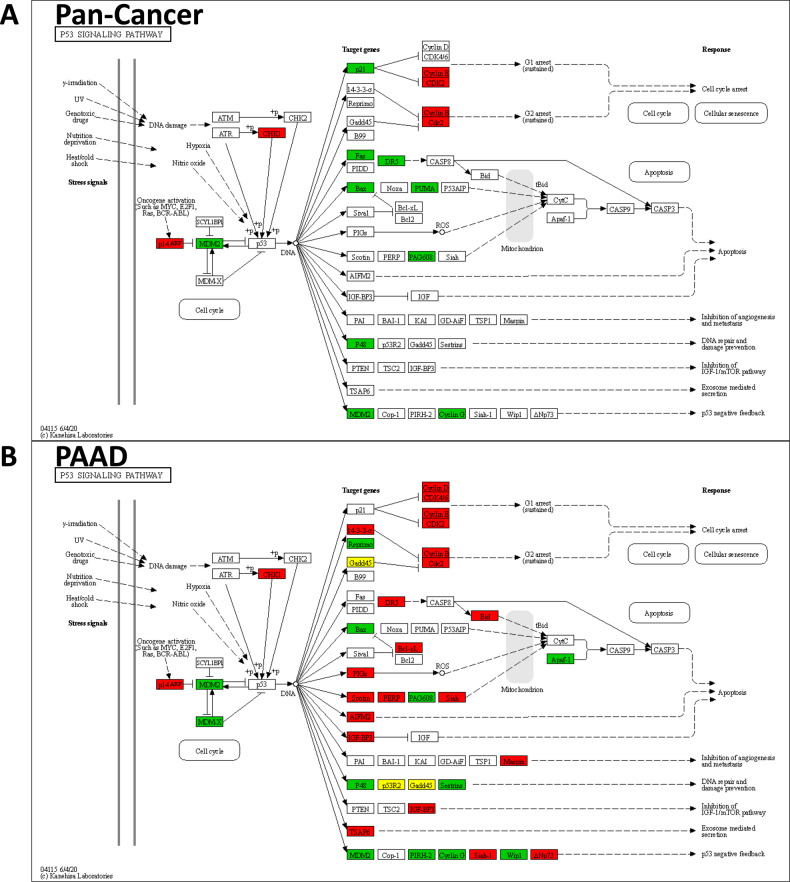
Significant gene expression changes between TP53mut and TP53wt tumors in the p53 signaling pathway. **A** pan-cancer consensus list. **B** pancreatic adenocarcinoma (PAAD). Red = overexpressed in TP53mut tumors. Gree*n* = underexpressed in TP53mut tumors. Yellow = over- and underexpressed genes.

KEGG pathway analysis (Table 1) revealed enrichment of the underexpressed genes of the consensus list in the p53 signaling pathway (9 genes, enrichment FC = 79.1), whereas the overexpressed genes were enriched for cell cycle (20 genes, enrichment FC = 17.6). In particular, the following genes of the consensus list in the p53 signaling pathway were underexpressed (Fig. 5A): (i) *CDKN1A* (p21) mediating cell cycle arrest, (ii) *FAS* (Fas), *TNFRSF10B* (DR5), *BAX* (Bax), *BBC3* (PUMA), and *ZMAT3* (PAG608) mediating apoptosis, (iii) *DDB2* (P48) mediating DNA repair and damage prevention, and (iv) *MDM2* and *CCNG1* (Cyclin G) mediating negative p53 feedback. *CHEK1* (checkpoint kinase 1), which phosphorylates and activates p53, was overexpressed. Similar gene expression patterns were observed for TP53mut tumors in most of the cancer types, but in PAAD we observed overexpression rather than underexpression of the apoptosis pathway (Fig. 5B). The distinct gene expression profile observed in PAAD could be due to the low tumor purity of the PAAD samples (median: 18%, [42]) and a pronounced contribution of the TME to the expression profile of the PAAD samples as a consequence.

**Fig. 6:**
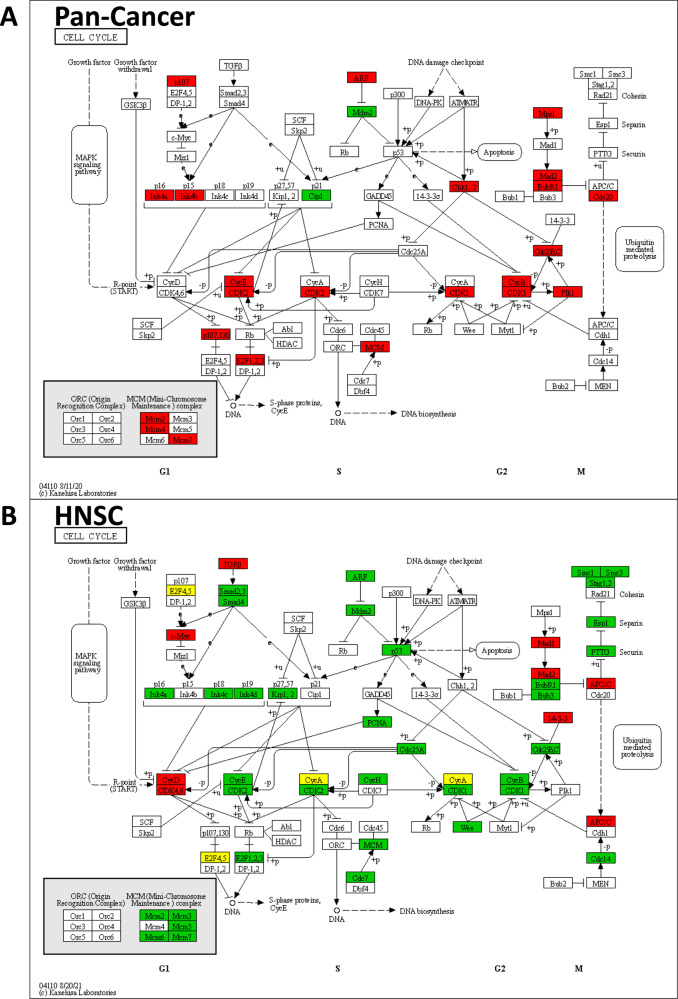
Significant gene expression changes between TP53mut and TP53wt tumors in the cell cycle pathway. **A** Pan-cancer consensus list. **B** Head and neck squamous cell carcinoma (HNSC). Red = overexpressed in TP53mut tumors. Gree*n* = underexpressed in TP53mut tumors. Yellow = over- and underexpressed genes.

Many of the overexpressed genes in the consensus list contribute to the regulation of the four phases (G1, S, G2, and M) of the cell cycle (Fig. 6A). In TP53mut tumors, we observed underexpression of *CDKN1A* (p21), and overexpression of *CCNE1* (CycE) and *CDK2*. Downstream of the cyclin-dependent kinases, we observed overexpression of *RBL1* (p107) and *E2F1* (Fig. 6A). Our results are consistent with a failure of TP53mut cells to arrest the cell cycle in the G1 phase mediated by missing induction of *CDKN1A* that does not inhibit the building of the *CCNE1*/*CDK2* complex as a consequence. In turn, the highly expressed *CCNE1/CDK2* complex is able to phosphorylate and inactivate *RB1* (Rb). The absence of activation of *RB1*, the binding partner of *E2F1*, as well as the observed overexpression of *E2F1*, are consistent with action of unbound E2F as transcription factor for downstream targets and transition to the S phase.

We observed overexpression of many genes regulating the S, G2, and M phases of the cell cycle (Fig. 6A). Among them were 17 genes in the list of DREAM complex targets according to Fischer et al. [39]: *BUB1B* (BubR1), *CCNB1* and *CCNB2* (CycB), *CCNE1* (CycE), *CDC20* (Cdc20), *CDC25C* (Cdc25C), *CDK1*, *CDK2*, *CHEK1* (Chk1), *E2F1*, *MAD2* (Mad2L1), *MCM2, 4, 7* (Mcm2,4,7), *PLK1* (Plk1), *RBL1* (p107), and *TTK* (Mps1). This observation is consistent with progression through the cell cycle and higher proliferation of TP53mut tumors compared to TP53wt tumors.

In contrast to almost all other cancer types, we observed underexpression instead of overexpression of many cell cycle genes in *TP53*-mutated HNSC (Fig. 6B). Human papillomavirus-negative (HPV-) compared to HPV-induced (HPV+) HNSC are known for distinct tumors biology, including a much higher prevalence of *TP53* mutations in HPV- HNSC [43]. In the study cohort, 80% of the HPV- tumors were TP53mut compared to only 26% of the HPV+ tumors. To investigate a potential confounding role of the virus infection, we stratified the analysis by HPV status (Suppl. Fig. S4). In the TP53mut tumors of HPV- HNSC, we observed underexpression of *CDKN1A* and overexpression of many cell cycle genes. By contrast, an expression pattern characterized by unchanged *CDKN1A* and underexpressed cell cycle genes was observed in the TP53mut tumors of HPV+ HNSC. Comparing the absolute level of *CDKN1A* expression (median levels) between cancer types, we found that TP53wt tumors of HPV- and HPV+ HNSC were among the cancer types with the highest *CDKN1A* expression (Suppl. Fig. S5). While the *CDKN1A* expression level was significantly lower in the TP53mut tumors for 20 of 26 cancer subtypes, including HPV- HNSC, it was numerically (non-significantly) higher in the TP53mut tumors of HPV+ HNSC. An unusual TP53-associated expression pattern of cell cycle genes in TP53mut tumors was also observed in CESC (Suppl. Fig. S5). As in HNSC, the prevalence of TP53mut was higher (53%) in HPV- CESC compared to HPV+ CESC (5%). In a stratified analysis of CESC, no significantly differentially expressed genes were observed, most probably because of low sample sizes for three of the four investigated groups (HPV- TP53wt: 9, HPV- TP53mut: 10, HPV+ TP53mut: 12). Altogether, these observations suggest a distinct role for *TP53* mutations in HPV infection-associated cancers.

### Immune tumor microenvironment in TP53mut tumors

**Fig. 7:**
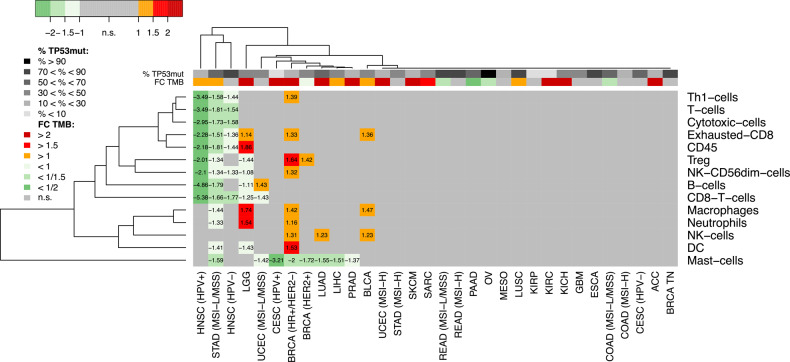
Immune cell population in the tumor microenvironment. Significant changes in immune cell abundance (FDR = 10%) between TP53mut and TP53wt tumors are coded in colors. Red = higher abundance in TP53mut tumors. Gree*n* = lower abundance in TP53mut tumors.

We analyzed the association of 14 specific immune cell populations in the TME with *TP53* mutations (Fig. 7). Because HPV+ tumors, tumors with microsatellite instability (MSI) as well as the molecular subtypes of breast cancer are associated with distinct characteristics of the immune TME, we stratified the analysis for 32 cancer subtypes. For 20 (63%) of these cancer subtypes, no significantly altered immune cell populations were detected. Among the remaining cancer subtypes, we noticed exclusively decreased immune cell populations in the TP53mut tumors of six subtypes, a mixed pattern of increased and decreased immune cell populations in four subtypes (HR+/HER2- BRCA, HER2+ BRCA, LGG, and LUAD), and exclusively increased immune cell populations in BLCA and PRAD. We observed a decrease of CD8+ T cell population in the TP53mut tumors of five subtypes (HPV- HNSC, HPV+ HNSC, LGG, MSI-L/MSS STAD, and MSI-L/MSS UCEC), while this cell population was not significantly altered in the remaining subtypes. Besides, we detected decreased regulatory T cells (Tregs) in the TP53mut tumors of HPV+ HNSC, MSI-L/MSS STAD, and LGG, while Tregs were increased in HR+ and HER2+ BRCA. In 15 (47%) of the cancer subtypes, we observed an increased TMB in TP53mut tumors, whereas in five (16%) cancer subtypes, we observed decreased TMB. In summary, we detected increased TMB in the TP53mut tumors of about the half of the cancer subtypes, but this sincreased TMB was not systematically associated with increase of immune cell infiltrations. A systematic influence of *TP53* mutations on the immune TME was observed only in a minority of cancer types.

## Discussion

We performed a comprehensive analysis of *TP53* mutations and the associated gene expression pattern in 8331 tumors of 24 major cancer types. In line with other studies [35, 44–46], we detected pronounced *TP53* mutation hotspots, most of them located in the DBD. The mutational hotspots showed a strong tendency to be shared across different cancer types. However, at the same time 53% of the hotspots showed significantly different prevalence between cancer types. For example, the prevalence of hotspot mutations was different in adeno- and squamous cell carcinoma of lung compared to cancers of the gastrointestinal tract. Recently investigated models including both mutational signatures and phenotype selection performed well in the prediction of mutation prevalence, while models including only one of these factors did not, suggesting that the pattern of *TP53* mutational hotspots is a consequence of both mutagenesis and selective forces that are active during tumor development [10]. In line with this result, the mutational processes operative in specific cancer types contributed to explaining the observed mixture of shared and individual hotspots. Examples included the ubiquitously operational clock-like process behind SBS1 and mutational processes active in specific cancer types, such as SBS4 and SBS24, associated with tobacco smoke and aflatoxin exposure.

We defined TP53 hotspot mutations by a minimun prevalence of of least 1% and presence in at least two tumors in at least one of the 24 investigated cancer types. For each of the investigated cancer types, less than 50% of all *TP53* mutations were hotspot mutations. For 14 of the 24 cancer types, <20% of all *TP53* mutations were hotspot mutations. The prevalence of a specific hotspot in a specific cancer type among all *TP53*-mutated cases was always less than 10%. These numbers indicate a low to medium prevalence of specific *TP53* mutations and advocate pooling of mutations to gain sufficient numbers of samples for statistical analysis.

Numerous experimental studies including systematic screens of synthetically generated variant libraries [9] reported distinct functional impact of different *TP53* mutation types including LOF, DN impact, and GOF [47]. Furthermore, studies of *TP53* germline mutations in animal models and cohorts of Li-Fraumeni syndrome patients revealed earlier cancer onset for specific *TP53* mutations [47–50]. By contrast, in the current study in a large pan-cancer cohort of human tumors, virtually no significant gene expression changes between different *TP53* mutation types were detected. This discrepancy could be explained as follows:

1. Some of the experimental studies of *TP53* mutation types are conducted models of tumor initiation, e.g., [48]. Potentially, the variability of cancer onset in these model systems and cohorts of Li-Fraumeni patients is due to differences in signaling during tumor initiation and the early phase of tumor development, but dissipate once the tumors are clinically detectable. Supporting this scenario, the second *TP53* allele was inactivated due to loss of heterozygosity or a second *TP53* mutation in more that 90% of the TCGA tumors [35]. Thus, while a DN effect of *TP53* mutations can be relevant during tumorigenesis, it is irrelevant for vast majority of tumors detected. Thus, the discrepancy between the reported functional impact of mutation type and the absence of differential expression in the TCGA cohort could be explained by a model in which the mutation type is only relevant in the early phase of tumor development before it becomes clinically apparent.
2. Analyzing specific types of *TP53* mutations drastically reduced the sample sizes. To enable comparability between different cancer types and between teh analysis of different *TP53* mutations types, we performed differential expression analyses for a fixed number of 10, 15, and 20 samples in each of the analyzed groups. Because of the limited number of samples, it is possible that the expression FCs between mutation types (compared to the variance of gene expression) were too low to reach statistical significance. However, many differentially expressed genes were detected in the analyses comparing TP53mut and TP53wt tumors. Thus, while we can not rule out that there are gene expression changes between different types of *TP53* mutations, the analyses show that - if they do exist - they are much weaker than the gene expression changes between TP53mut and TP53wt tumors. Furthermore, as an approach to enhance the statistical power, summary of significances across all cancer types did not also lead to significant results for differential expression between different mutation types.
3. *TP53* predominantly acts as a transcription factor that binds to specific DNA response elements and exerts its functions via transcriptional regulation [51, 52]. Although the most prevalent DBD mutations largely abrogate the regulation of canonical p53 wildtype target genes, mutant p53 proteins can exert transcriptional activities by interacting with other transcription factors or chromatin-modifying complexes [53]. In turn, these effects strongly depend on the factors expressed, therefore differ between cell and tissue types and genetic context, and may be too diverse to be detectable with the available number of tumors per mutation. Moreover, many pro-metastatic properties of mutant p53 proteins operate at a non-transcriptional level, for example, by modulating protein biosynthesis and secretion [54, 55], so the biological differences observed in experimental systems and during the initiation of human tumors might be hidden when analyzing mRNA expression profiles.

In the current study, we observed shared TP53mut-associated gene expression patterns across many cancer types and carved out a consensus list of 210 genes that were differentially expressed in at least 16 of 24 cancer types. The overexpressed genes of the list were strongly enriched for G2/M cell cycle genes, while the underexpressed genes were strongly enriched for direct targets of p53 and apoptosis genes in accordance with the literature [40, 56]. Enrichment for G2/M cell cycle genes was found for all investigated cancer types with the only exception of CESC and HNSC cohorts, which included a substantial proportion of HPV-positive tumors (93% and 18%). The HPV E6 and E7 proteins bind *TP53* and *RB1*, respectively, and inactivate the functions of these tumor suppressor genes [57, 58]. Both *RB1* and *TP53* act as negative cell cycle regulators, explaining the anomalous *TP53*-associated expression patterns observed in cohorts that include HPV-induced tumors. When restricting to HPV- HNSC, the typical *TP53*-associated expression pattern was observed.

The role of *TP53* mutations in response to immune checkpoint blockade (ICB) is controversial. In a recent meta-analysis combining six whole exome sequencing (WES) data sets across cancer types, *TP53* mutations were a negative predictor of ICB response [59]. By contrast, *TP53/KRAS* co-mutated non-small cell lung cancer patients benefited from PD-L1 blockage in comparison to docetaxel, while *KRAS*-mutant patients without additional *TP53* mutation did not [60]. A distinct immunoregulatory program was uncovered in *TP53*/*KRAS* co-mutated pancreatic ductal adenocarcinoma [61]. In models of TP53mut triple-negative breast cancer restoration of *TP53* activity sensitized for blockade of the PD-L1/PD1 axis [62]. While it could be expected that inactivation of *TP53* that acts as guardian of the genome would increase TMB and the number of neoantigens, the current study showed that the immune TME was unaltered in TP53mut tumors of the majority of cancer types. Upregulated immune cell populations were detected only in very few cancer types and never included CD8+ T cells. Decreased CD8+ T cell populations were detected in the TP53mut tumors of HNSC, LGG, MSI-L/MSS STAD, and MSI-L/MSS UCEC. Altogether, our study suggests that the immune TME is modified in a *TP53* status-dependent manner in specific cancer types. Further studies are warranted to investigate the implications for the guidance of immune therapies.

Many of the genes of the consensus list (52%) were related to the cell cycle. In line with the observation that the expression levels of these genes were prognostic in several cancer types, the cell cycle machinery represents a target for established drugs and agents under development [63, 64]. Among these, CDK4/6 inhibitors are approved to treat certain types of hormone receptor-positive, HER2-negative breast cancer in combination with endocrine therapy. *CDK4* and *CDK6* were not included in the consensus list, but overexpressed in the TP53mut tumors of respectively 14 and 10 cancer types (just below the threshold 16 for inclusion in the consensus list). *CDK1* and *CDK2* were included in the consensus list and corresponding inhibitors and are currently tested in phase I, II and III trials. *CCNE1* (clinical studies), as well as *ADAM17* and *CDC20* (preclinical), are additional examples of genes on the consensus list currently being considered for therapeutic invention [65–67]. Correlation of the expression level of many of the currently investigated target genes with TP53 mutation status supports the view that *TP53* mutations should by further investigated as a predictive marker to select patients for cell cycle targeting therapies.

Although we analyzed one of the largest comprehensively molecular characterized cancer cohorts available, small sample sizes in the analysis of specific *TP53* mutations in specific cancer types represent the main limitation of the current study. As gene expression patterns differ strongly between cancer types, pooling of cancer types would lead to a drastic increase in variance and would be ineffective to overcome this limitation. As further limitation, the study was focused on operable, early-stage tumors (TCGA) and on the molecular layer of gene expression. As several studies support a distinct role of different *TP53* mutations in metastatic dissemination [68–70], analysis of further cohorts of advanced-stage tumors is warranted. Furthermore, inclusion of additional molecular layers, in particular of the phosphoproteome, would be beneficial.

This is the first study to comprehensively analyze the effect of specific *TP53* mutation types on mRNA expression patterns across cancer types. Because we detected virtually no mutation type associated alterations, we pooled *TP53* mutation types for comparison to TP53wt tumors. We extracted list of 210 genes that were differentially expressed between TP53mut and TP53wt tumors in two-thirds or more of the 24 cancer types. We also performed differential gene expression analysis for each cancer type followed by gene set enrichment analyses and uncovered impaired biological processes in TP53mut tumors of each entity. Analysis of specific immune cell populations showed an influence of *TP53* mutations on the composition of the immune TME for 12 of the 32 investigated cancer subtypes. The analysis of a large cohort of human tumors complements results from experimental studies and supports the view that *TP53* mutations should be further evaluated as predictive markers for cell cycle targeting therapies, immunotherapies, and others.

## Material and methods

### Study cohort

We included 8331 tumors and 24 cancer types from the TCGA for which mutation calls, mRNA expression data were available, and at least seven TP53mut tumors per entity were available (Suppl. Table S1). Mutation calls (mc3.v0.2.8.PUBLIC.maf.gz) and expression data (EBPlusPlusAdjustPANCAN_IlluminaHiSeq_RNASeqV2.geneExp.tsv) were downloaded from the Genomics Data Commons (GDC) webpage [71]. Tumor mutational burden (TMB) was calculated as the total of missense mutations.

### Classification of mutations

In the first step, tumors were classified as *TP53*-mutated (TP53mut) or *TP53*-wildtype (TP53wt). Tumors harboring missense, nonsense, splice site, translation site, or non-stop mutations or frameshift or in-frame indels were classified as TP53mut. Tumors without mutations, with synonymous mutations, or with mutations in the gene flanks, introns, UTRs, splice regions, or intergenic regions were classified as TP53wt.

In the second step, *TP53* mutations were classified as GOF, LOF, DN, and not DN mutations (Suppl. Fig. S1 and Suppl. Table S3). First, mutations were annotated using The TP53 Database (version R20) [72], with mutations annotated as both GOF and LOF being classified as GOF. Second, mutations not present in the TP53 Database were either classified as LOF, if there was evidence for truncation (nonsense mutations, frameshift indel, splice site mutations, transcription start site mutations, or non-stop mutations) or as a variant of unknown significance (VUS), if not.

### Statistical analysis and visualization

Statistical analyses and graphics generation were performed by using R (version 4.1.2) and RStudio Desktop (version 2.0.443) [73].

### Analysis of mutational hotspots

A lollipop diagram of the distribution of the detected *TP53* variants was created with the MutationMapper at the cBioPortal [74, 75]. The prevalence of the recurrent variants (detected in at least two tumors and with at least 1% prevalence in at least one cancer type) was visualized in a heatmap using the R package heatmaply [76]. The recurrent mutations were tested for differential prevalence in the cancer types using the functions prop.test of the R package binom [77] and the *p-*values were corrected for multiple testing using the Benjamini-Hochberg (BH) method. A set of hotspots with different prevalences of *TP53* mutations in different cancer types was compiled controlling the false discovery rate (FDR) at 10%. The prevalence of mutational hotspots was reported together with 95%-confidence intervals calculated with the Clopper-Pearson method.

### Differential gene expression analysis

Sample-normalized gene expression data (upper quartile normalization) were transformed to the *log2* scale. The significance of differential gene expression was assessed using the Wilcoxon rank-sum test. Exact *p-*values were corrected for multiple testing using the BH method. Lists of differentially expressed genes were extracted, controlling the FDR at 10%.

We sought to compare gene expression patterns between tumors with different types of *TP53* mutations as well as TP53wt tumors. To ensure comparability, differential gene expression analyses were performed with a fixed sample size (*n* = 10, 15, and 20), and tumors of each cancer type and mutation type were randomly selected for the corresponding analyses. First, we performed comparisons between different *TP53* mutation classes (R175, R248, R273, Hotspots, LOF, GOF, DN, and non-DN). The *TP53* mutation class “Hotspots” included the top ten most frequent missense mutations: R175H, R273C/H, R248Q/W, R282W, Y220C, G45S, H179R, and V157F. Second, we compared the different *TP53* mutation classes to TP53wt tumors.

Because we did not detect characteristic gene expression patterns associated with specific *TP53* mutations, we analyzed the differential gene expression between TP53mut and TP53 tumors irrespective of the mutation type. The *p-*values of the 24 cancer types were summarized to pan-cancer *p-*values using Fisher’s method [78]. A 210-gene consensus list was created including all genes with significant expression changes (raw *p* < 0.05) in at least 16 cancer types. Fold changes (FCs) of the significantly differentially expressed genes were visualized as heatmap with hierarchical clustering using the Manhattan metric to measure the distance between cancer types and between genes and the average linkage method to measure the distance between clusters. Clusters of cancer types were tested for different *TP53* mutation prevalence using the Kruskal-Wallis rank sum test.

### Survival analysis

Analysis of progression-free interval (PFI) and overall survival (OS) was performed using the TCGA-Clinical Data Resource (CDR) Outcome (TCGA-CDR-SupplementalTableS1.xlsx) [71]. First, we compared the survival of TP53mut and TP53wt tumors of each cancer type. Second, we analyzed the association of the expression level (cutpoint: median) of each gene in the consensus list with survival using Cox regression.

### Functional analysis

The Molecular Signatures Database (MSigDB v7.5.1) was downloaded from the GSEA web page [79] and imported using the R package XML [80]. We analyzed the following MSigDB catalogs: H (Hallmarks, *n* = 50), C2 (Curated Gene Sets: Martin Fischer and Kyoto Encyclopedia of Genes and Genomes (KEGG), *n* = 192), and C5 (Gene Ontology (GO), *n* = 2). The significance of the enrichment of functional categories in the catalogs was assessed using the Fisher test and corrected for multiple testing using the BH method at FDR of 10%. The strength of enrichment or depletion of a functional category in the consensus gene list was quantified by the enrichment fold change,

$$FC = \frac{{k/K}}{{n/N}}$$

with *k* being the number of genes in the gene list annotated for the functional category, *K* the total number of genes in the gene list, *n* the total number of genes in the functional category and *N* the total number of the genes in the functional catalog. Significant over- or underexpression of genes in signaling pathways was visualized using KEGG Tools [81].

## Supplementary information

Supplemental Figures

Supplemental Table S1

Supplemental Table S2

Supplemental Table S3

Supplemental Table S4

## Data Availability

The TCGA data analyzed in this study are publicly available from the NIH Genomic Data Commons as stated in the “Methods” section.

## Code availability

The R code is available from the authors upon request.
