# Supplementary material for: Homogenous TP53mut-associated tumor biology across mutation and cancer types revealed by transcriptome analysis
Source: Cell Death Discov. 2023 Apr 14;9:126. doi: 10.1038/s41420-023-01413-1 (PMC10104808; doi:10.1038/s41420-023-01413-1)
Supplement: Supplementary file 1 — Supplemental Figures [file 41420_2023_1413_MOESM1_ESM.pdf]

Supplemental Material:

Homogenous TP53mut-associated tumor biology across mutation and cancer types revealed by transcriptome analysis

1. **Figures:**
   1) **S1: Functional Classification of TP53 mutations.**
   2) **S2: Gene expression patterns associated with GOF, LOF, DN, and non-DN TP53 mutations.**
   3) **S3: Prognostic impact of TP53 mutations and of the gene expression of the 210 genes in the consensus list.**
   4) **S4: Differential gene expression related to the regulation of the cell cycle in head and neck squamous cell carcinoma (HNSC) stratified by HPV infection status.**
   5) **S5: Significant differential gene expression related to the regulation of the cell cycle in cervical squamous cell carcinoma (CESC).**
   6) **S6: Gene expression levels of CDKN1A (p21), MDM2, CDKN2A in 26 cancer types.**
2. **Tables:**
   1) **External File: Table S1: TCGA cancer types included in (A, n=24) and excluded (B, n=9) from the study. *supplement_table_1_numbers_samples_mutclasses.xls***
   2) **External File: Table S2: Prevalence of the 59 most recurrent TP53 mutations in 24 cancer types. Mutations with a prevalence of at least 1% in at least one of the cancer types were included in the analysis. *supplement_table_2_pvalues_upper_lower_prevalence_mutations_mutTP53_binom.xls***
   3) **External File: Table S3: Classification of the TP53 mutations in the TCGA data set. The table includes the information on the type of TP53 mutation (LOF, GOF, DN, non-DN, or wt) for each of the TCGA tumors. *supplement_table_3_TP53muts_classification.xls***
   4) **External File: Table S4: Consensus list of 210 differentially expressed genes between TP53mut and TP53wt tumors. The table includes the statistical values for the expression of each gene (fold change and *p*-value). *supplement_table_4_consensus_list_genes.xls***

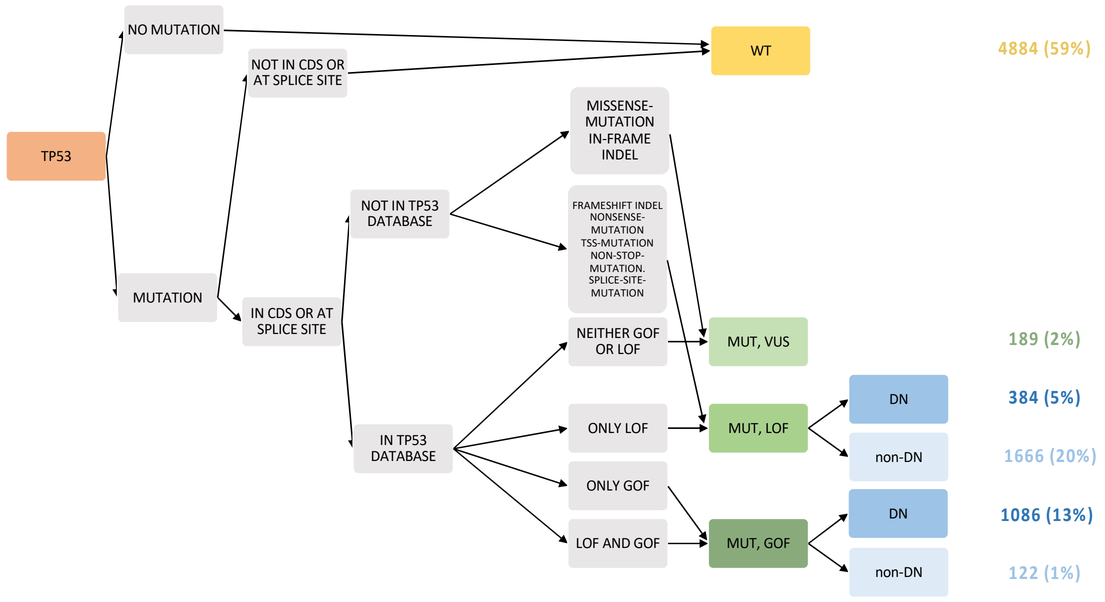

Figure S1: Functional Classification of TP53 mutations. Non-synonymous and silent mutations were classified as wildtype (TP53wt). The TP53 Database was used to classify TP53 mutations (TP53mut) according to their functionality (LOF, GOF, DN, and non-DN). Variants not available in the TP53 Database were classified manually. The study cohort included a total of 3447 TP53mut tumors, of which 59% were classified as LOF, 35% as GOF, and the remaining 5% as VUS. Most LOF tumors were non-DN (81%), as most GOF were DN (90%).

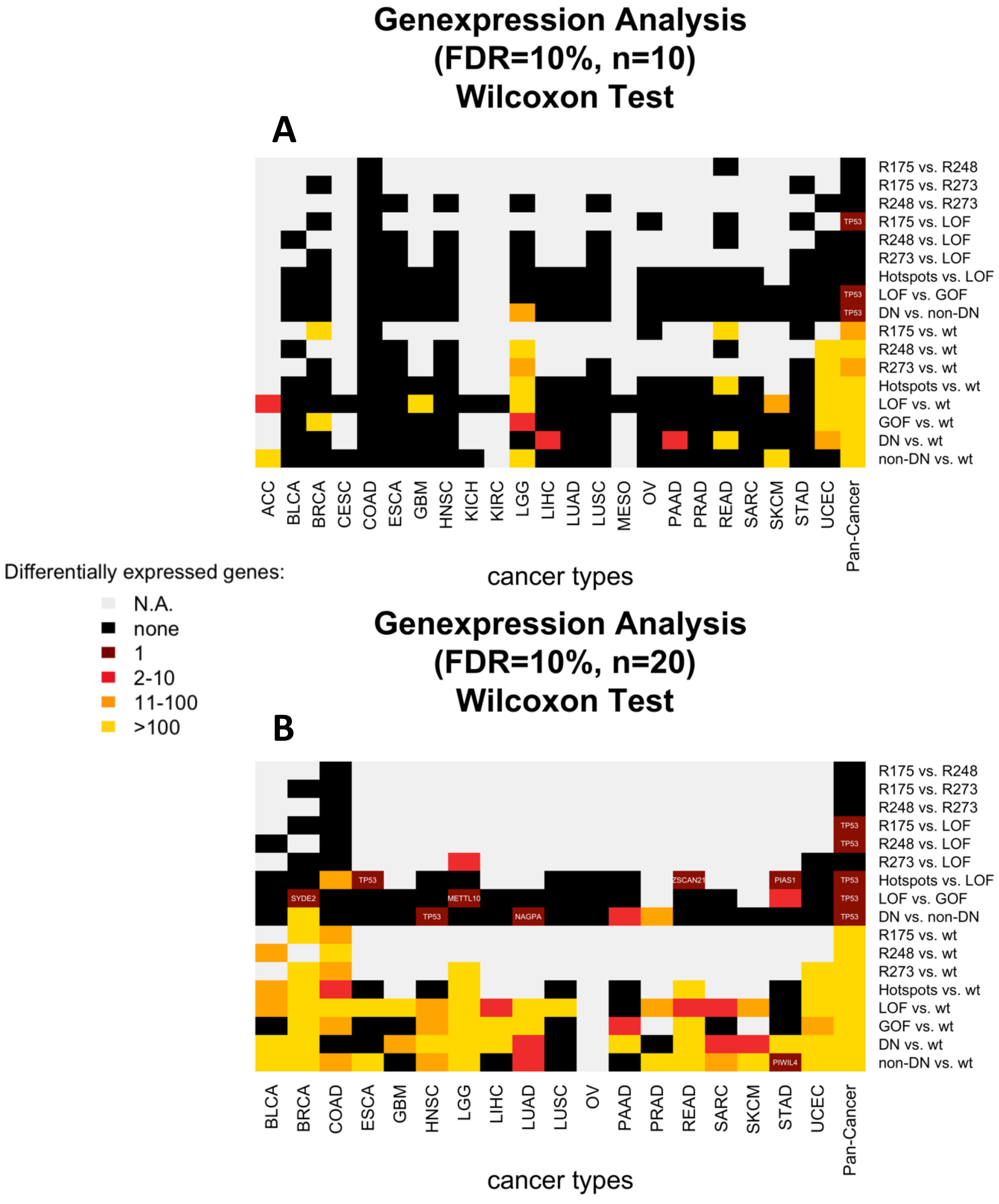

Figure S2: Gene expression patterns associated with GOF, LOF, DN, and non-DN TP53 mutations. Same analysis as in Figure 3, but for 10 vs. 10 samples **(A)** and for 20 vs. 20 samples **(B)**. For the pan-cancer analysis, the results in the specific cancer were summarized using Fisher's method. None = no significantly expressed genes detected, N.A. = analysis not possible (insufficient number of samples).

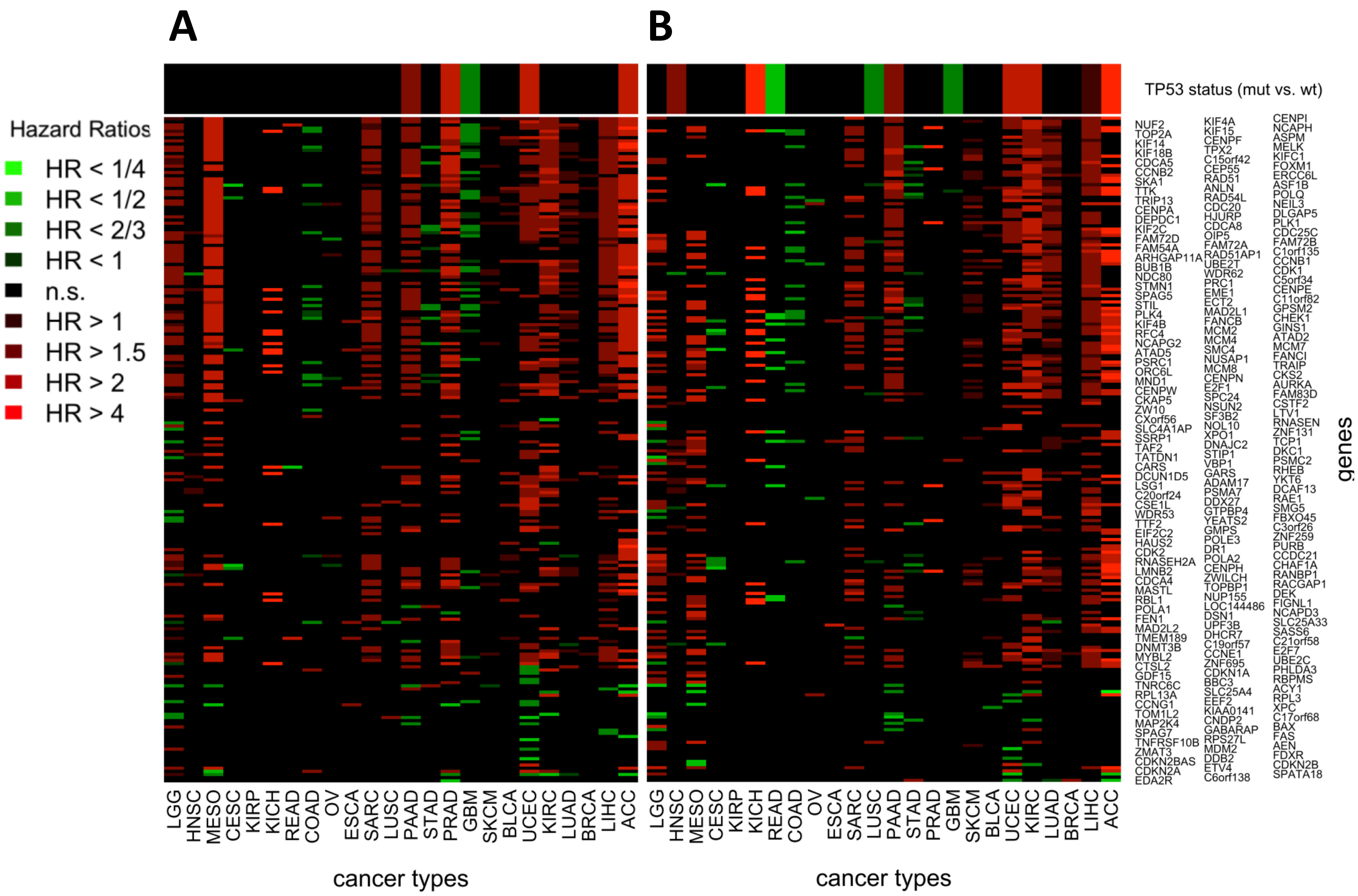

Figure S3: Prognostic impact of TP53 mutations and of the gene expression of the 210 genes in the consensus list. Analysis of PFI **(A)** and OS **(B)**. Hazard Ratios (HR) between TP53mut and TP53wt tumors, as well as between tumors with high gene expression and tumors with low gene expression (cutpoint: median of gene expression). Significant survival differences (FDR=10%) are coded in red for association of TP53 mutations/of high gene expression with unfavorable prognosis) and in green for association of TP53 mutations/high gene expression with favorable prognosis.

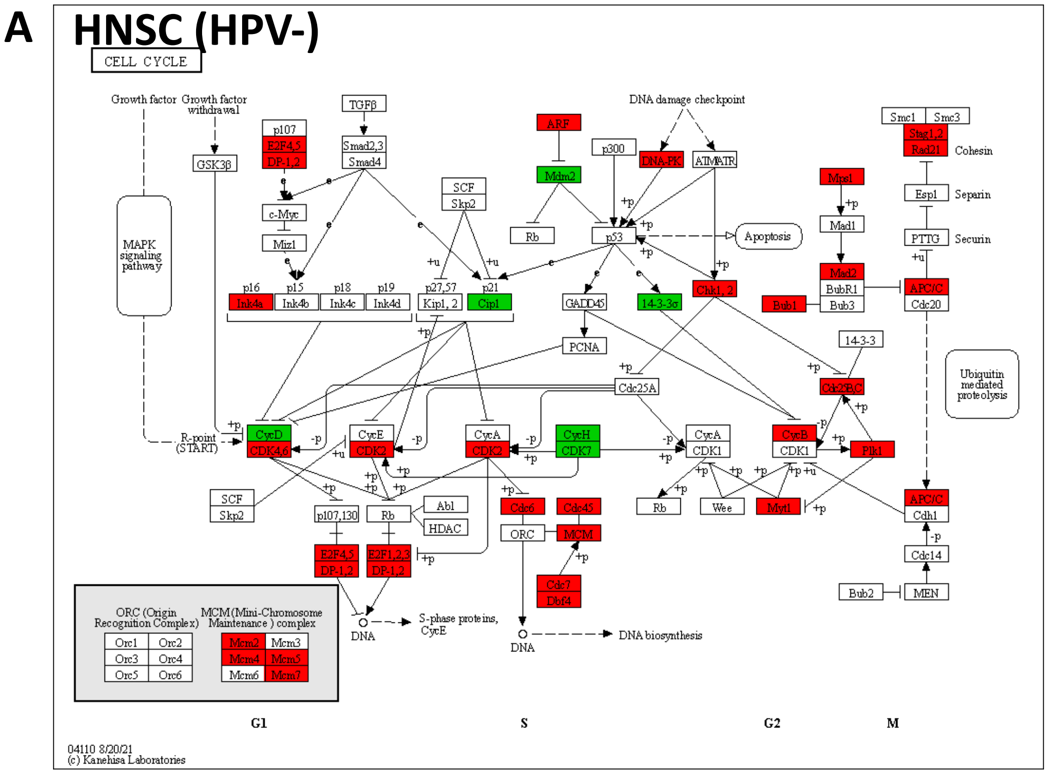

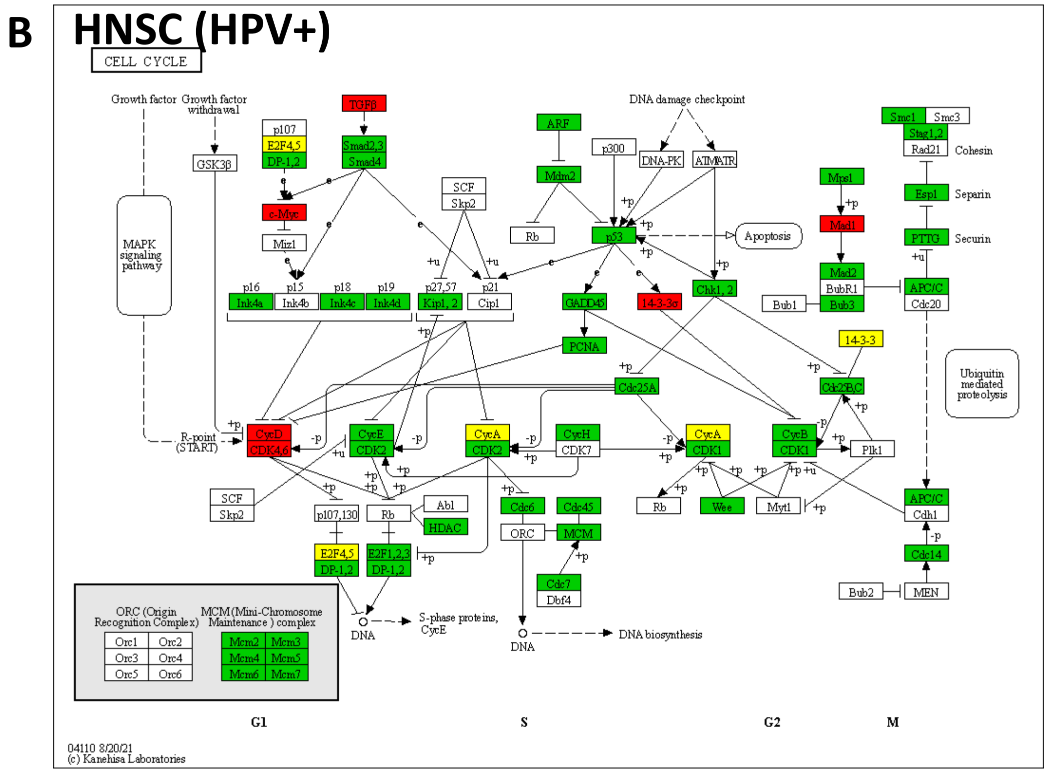

Figure S4: Differential gene expression related to the regulation of the cell cycle in head and neck squamous cell carcinoma (HNSC) stratified by HPV infection status. A HPV- HNSC **B** HPV+ HNSC. Red = overexpressed in TP53mut tumors. Green = underexpressed in TP53mut tumors. Yellow = over- and underexpressed genes.

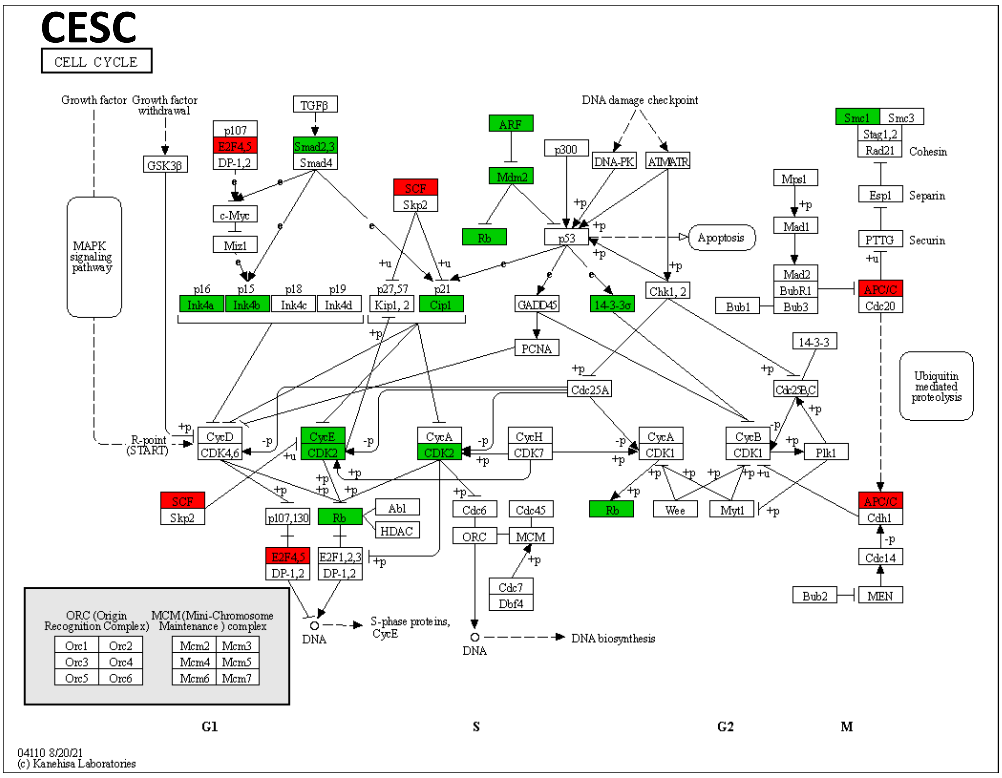

Figure S5: **Significant differential gene expression related to the regulation of the cell cycle in cervical squamous cell carcinoma (CESC).** Red = overexpressed in TP53mut tumors. Green = underexpressed in TP53mut tumors. Yellow = over- and underexpressed genes.

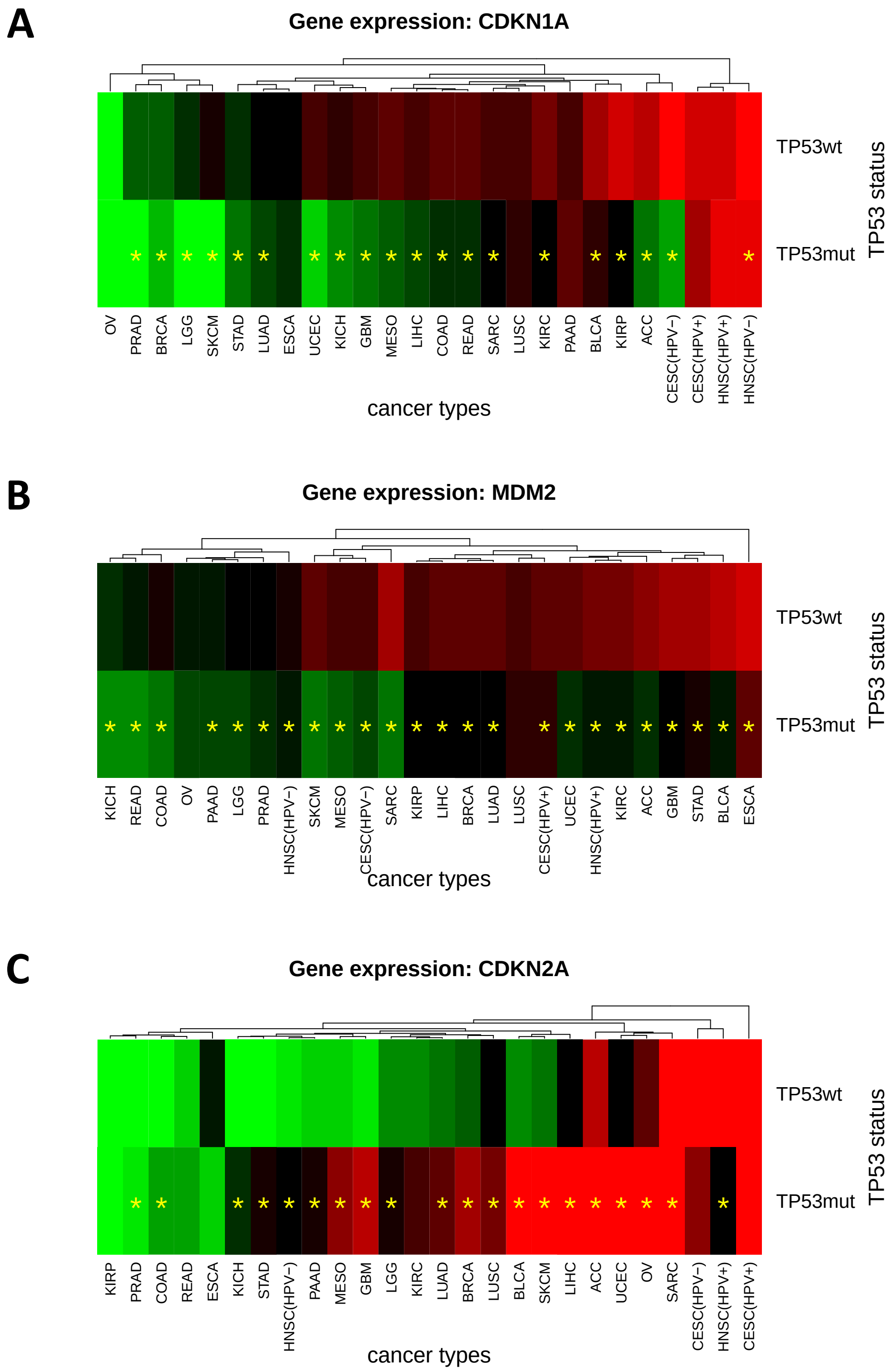

Figure S6: Gene expression levels of (A) CDKN1A (p21), (B) MDM2 and (C) CDKN2A (ARF) in 26 cancer types. Colors encode the mean gene expression level in the TP53mut and the TP53wt tumors of each cancer type. Red colors encode gene expression above the median expression level across all cancer types, and green color encodes gene expression below the median expression level across all cancer types. Stars indicate differential expression between TP53mut and TP53wt tumors.
